# Evoking Apparent Moving Sensation in the Hand via Transcutaneous Electrical Nerve Stimulation

**DOI:** 10.3389/fnins.2020.00534

**Published:** 2020-06-18

**Authors:** Alessia Scarpelli, Andrea Demofonti, Francesca Terracina, Anna Lisa Ciancio, Loredana Zollo

**Affiliations:** Research Unit of Advanced Robotics and Human-Centred Technologies, Università Campus Bio-Medico di Roma, Rome, Italy

**Keywords:** sensory feedback restoration, transcutaneous electrical nerve stimulation, apparent moving sensation, upper limb prostheses, slippage

## Abstract

The restoration of sensory feedback in amputees plays a fundamental role in the prosthesis control and in the communication on the afferent channel between hand and brain. The literature shows that transcutaneous electrical nerve stimulation (TENS) can be a promising non-invasive technique to elicit sensory feedback in amputees, especially in the lower limb through the phenomenon of apparent moving sensation (AMS). It consists of delivering a sensation that moves along a specific part of the body. This study proposes to use TENS to elicit tactile sensations and adopt AMS to reproduce moving sensations on the hand, such as those related to an object moving in the hand or slipping upward or downward. To this purpose, the developed experimental protocol consists of two phases: (i) the mapping of the evoked sensations and (ii) the generation of the AMS. In the latter phase, the pulse amplitude variation (PAV), the pulse width variation (PWV), and the interstimulus delay modulation (ISDM) methods were compared. For the comparative analysis, the Wilcoxon–Mann–Whitney test with Bonferroni correction (*P* < 0.016) was carried out on the success rate and on the ranking of methods expressed by the subjects. Results from the mapping protocol show that the delivered sensations were mostly described by the subjects as almost natural and superficial tingling. Results from the AMS protocol show that, for each movement direction, the success rate of ISDM method is higher than that of PWV and PAV and significantly higher than that of PAV for the ulnar-median direction. It recreates an AMS in the hand that effectively allows discriminating the type of sensation and distinguishing the movement direction. Moreover, ISDM was ranked by the subjects as the favorite method for recreating a well-defined and comfortable moving sensation only in the median-ulnar direction. For the ranking results, there was not a statistically significant difference among the methods. The experiments confirmed the good potential of recreating an AMS in the hand through TENS. This encourages to push forward this study on amputees and integrate it in the closed-loop control of a prosthetic system, in order to enable full control of grasp stability and prevent the objects from slippage.

## Introduction

Upper limb loss is a traumatic event for a human being from a functional and social viewpoint (Ciancio et al., 2016; Cordella et al., 2016). Upper limb prostheses want to replace in the amputee the lost functions and contribute to improve people quality of life. Commercially available hand prostheses use, for hand grasping, an open loop control strategy that does not involve the user in the control loop of the device. Despite that current open loop control strategies have shown good results (Cordella et al., 2014), the amputee can only rely on visual feedback, and this increases the cognitive efforts due to the lack of sensory feedback during manipulation tasks.

For that reason, new approaches aim to insert the user in the control loop of robotic system for upper limb rehabilitation and for prosthetic application. These techniques would lead to monitor the user state and accordingly change the robot behavior (Papaleo et al., 2013). Closed-loop devices for prosthetic application overcome open-loop device limitations: they can improve the performance of the tasks, guarantee a better usability, and a higher embodiment (Wright et al., 2016). Current studies aim to restore the bidirectional communication between the nervous system and the user through closed-loop devices, in order to improve the performance of the motor control and include the user in the loop through the restoration of sensory feedback (Antfolk et al., 2013; Ciancio et al., 2016; Cordella et al., 2016; D’Anna et al., 2017).

It has been demonstrated that invasive interfaces with peripheral nervous system (PNS) [which require surgery to be implanted (Navarro et al., 2005)] are an efficient method to restore a bidirectional communication between the user and the prostheses (Antfolk et al., 2013). Although they allow obtaining promising results, such as the selectivity of the elicited sensation, the discrimination of the hand areas, and the possibility to restore an artificial sensation similar to the real one, they present some disadvantages related to invasiveness, such as the surgery, the fibrotic reaction, and the weak long-term stability of the implant feedback (Raspopovic et al., 2014; Tan et al., 2014; Oddo et al., 2016; D’Anna et al., 2019; George et al., 2019; Zollo et al., 2019).

Different types of non-invasive interfaces have been tested in several studies to close the patient in the prosthesis control loop, e.g., vibrotactile (Cipriani et al., 2011), mechanical (Kim and Colgate, 2012), auditory (Gonzalez et al., 2012), or electrical interfaces. However, they have many drawbacks related to a high cognitive burden that also leads to increase in the response time, a low selectivity in the recognition of the elicited sensation, a low discrimination capabilities of hand areas, a very unnatural sensation, and a long phase of training (Kaczmarek et al., 1991; D’Anna et al., 2017).

Evidence suggests that transcutaneous electrical nerve stimulation (TENS) can be a promising technique as non-invasive closed-loop interface (Johnson and Bjordal, 2011; Chai et al., 2015). TENS uses superficial electrodes placed on the skin to electrically stimulating the PNS and evoke tactile sensation (Chai et al., 2015).

The literature shows that TENS can reduce painful conditions (Johnson and Bjordal, 2011), phantom pain, and stump pain caused by amputation (Johnson et al., 2015). It has also been demonstrated that electrocutaneous stimulation of the median and ulnar nerve can enable the closed-loop control of a prosthesis (Antfolk et al., 2013) and deliver touch and pain sensations (Osborn et al., 2018). This method is safe; it has low energy consumption and high response rate compared to other techniques (Antfolk et al., 2013; D’Anna et al., 2017; Osborn et al., 2017; Vargas et al., 2019).

Recently, a novel feedback principle has been introduced, named apparent moving sensation (AMS). It consists of delivering a sensation that moves along a specific part of the body. The AMS exploits a psychological phenomenon called *tactile phi phenomenon*, which describes a phantom sensation between two stimuli that are simultaneously presented in adjacent locations on the human skin (Pfeifer et al., 2010; Lauretti et al., 2017). If the intensities of the two stimuli are the same, the phantom sensation is felt in the midpoint between their locations. On the other hand, if the two stimuli have different intensities, the phantom sensation is felt around the location of the stimulus with the higher intensity. Therefore, properly modulating the two intensities, the sensation can be moved between the two stimuli locations (Lauretti et al., 2017). In the literature, AMS was applied through TENS to lower limb to make amputees realize how the position of the center of pressure (CoP) changes during gait (Rahal et al., 2009; Pfeifer et al., 2010; Seps et al., 2011; Pagel et al., 2016).

This study considers the aforementioned advantages of non-invasive techniques and focuses on the application of TENS to upper limb amputees in order to elicit tactile sensations in the hand. TENS technique enables a closed-loop control of the prosthesis.

In this study, TENS is aimed to elicit tactile sensations in well-defined hand areas and adopt AMS on the hand in order to reproduce moving tactile sensations on different areas of the hand. This strategy can provide the user with force information when grasping objects and also moving sensations (such as slippage) during object manipulation. To this purpose, an experimental validation has been carried out on nine healthy subjects. The experimental protocol consists of two phases: (i) the mapping of the evoked sensations and (ii) the generation of the AMS on the subjects’ hands. In the latter phase, the pulse amplitude variation (PAV), the pulse width variation (PWV), and the interstimulus delay modulation (ISDM) have been compared.

The paper is organized as follows. *Materials and Methods* describes the experimental setup, mapping protocol, and AMS strategy. *Results* reports results of the experimental session. Finally, *Discussion* discuss the results and draws the conclusions.

## Materials and Methods

### Experimental Setup

Nine healthy subjects (four males and five females) with a mean age of 25.2 ± 3.1 years were recruited for the study. All subjects had no known neurological disorders and no previous experiences with TENS. The study was authorized by the Ethic Committee of Campus Bio-Medico University of Rome in accordance with the Helsinki Declaration; the main aspects of the study were explained to the subjects, and they signed an informed consent.

**FIGURE 1:**
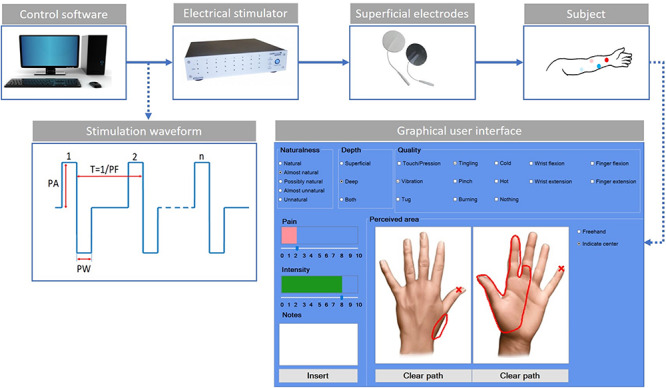
Scheme of the experimental setup. The control software sends the features of the stimulation waveform to the electrical stimulator. The stimulation waveform used in the study is the symmetric biphasic square wave, whose parameters are the pulse amplitude (PA), the pulse width (PW), and the pulse frequency (PF). The current is applied through the use of superficial electrodes to the user’s skin. The red dots indicate the electrodes for the median stimulation, while the blue ones are for the ulnar stimulation. The light color is used for the active electrodes and the shaded color for the passive electrodes. The custom-developed graphic user interface is used by the subjects to indicate the main characteristics of the evoked sensations.

The used experimental setup (Figure 1) was composed of an electric stimulator, a proprietary control software of the stimulator, four superficial electrodes, and a graphic user interface.

The electrical stimuli were delivered by the multichannel fully programmable stimulator (STG4008, Multichannel System MCS GmbH, Reutlingen, Germany). It has eight independent channels and allows stimulating more than one site simultaneously and independently. The proprietary software (MC_Stimulus II) of the stimulator allows generating arbitrary waveforms for each channel.

The subject sat in a chair in a comfortable position with his/her left arm placed on a table; then, the targeted skin area was cleaned with alcohol. Four commercial autoadhesive, circular, and superficial electrodes (TensCare) with a diameter of 25 mm were applied on the subject’s epidermidis and were used to selectively stimulate the subject nerves.

Finally, a custom-developed graphic user interface implemented in C# was used to record the main features of the elicited sensations (Figure 1). For each trial, the subject was asked to indicate the naturalness, the depth, the quality of the intensity, and the pain of the sensation. The naturalness of the sensation was assessed using a five-point scale, in which the lowest value means that the subject felt an unnatural sensation and the highest a natural one. Between these two values, other three options have been considered (Flesher et al., 2016; Kim et al., 2018; Petrini et al., 2019; Zollo et al., 2019). Therefore, the naturalness was assessed using the following options: natural, almost natural, possibly natural, almost unnatural, and unnatural. The depth was assessed choosing between superficial, deep, or both. The quality was assessed using the following choices: touch/pression, vibration, tug, tingling, pinch, burning, cold, hot, wrist flexion, wrist extension, finger flexion, finger extension, and nothing. The intensity and/or the pain of the sensation were reported in a scale from 0 to 10. The subject had to indicate the location of the sensation using two pictures representing the dorsal and palmar side of the hand (Figure 1).

The symmetric biphasic square wave was found to be the most used since it was shown to be able to elicit a more comfortable sensation among the others (Chai et al., 2015; D’Anna et al., 2017; Li et al., 2018; Shin et al., 2018; Vargas et al., 2019). The stimulation parameters taken into account are shown in Figure 1: pulse amplitude (PA), pulse width (PW), and pulse frequency (PF). No interphase delay (ID) has been used.

The experimental setup in Figure 1 was used both for mapping and AMS protocol.

### Mapping of the Elicited Sensations

**FIGURE 2:**
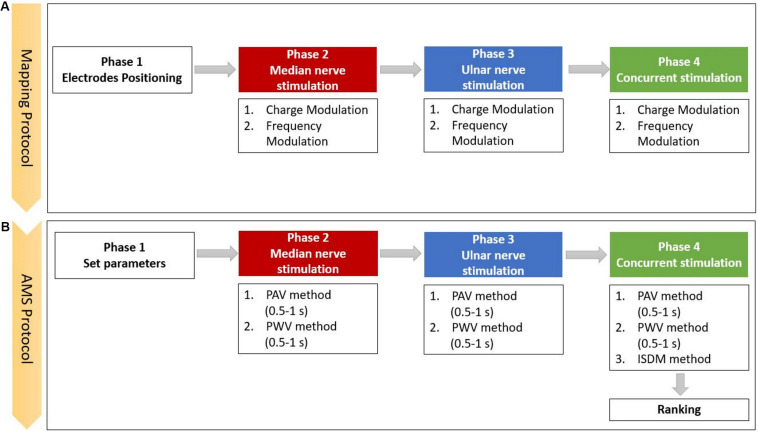
Scheme of the experimental protocol, which is composed of the **(A)** mapping and the **(B)** apparent moving sensation (AMS) protocols (PAV, pulse amplitude variation; PWV, the pulse width variation; and ISDM, interstimulus delay modulation methods).

The mapping protocol (Figure 2A) was composed of four phases: the electrodes positioning, the median nerve, the ulnar nerve, and the concurrent stimulation phases. In the concurrent phase, both nerves were stimulated simultaneously. Each stimulation phase included charge modulation and frequency modulation. For each trial, the subject had to report the characteristic of the sensation in the graphic interface shown in Figure 1. At the end of each phase, the specific reported characteristics of the evoked sensation were used to set the stimulation parameters for the successive phase.

The first part of the mapping protocol was aimed to identify the optimal position for the two pairs of electrodes. They had to be positioned upon the skin along the superficial path of the median and ulnar nerves in order to stimulate the underlying nerve and elicit a sensation in the areas of hand and fingers innervated by those nerves. The optimal positioning was identified by varying the location of each pair of electrodes. During the three phases of median, ulnar, and both nerves stimulation, the PW and PF parameters were modulated, and the perceived sensations were recorded.

The minimum and maximum values of pulse amplitude of both nerves were defined using the following stimulation parameter: the PW and the PF were fixed, respectively, to 500/600 μs and 500/600 Hz for the median/ulnar nerves, whereas the PA was incremented from 1 mA with a step of 0.1 mA. PA_min_ is the first value of PA at which the subject reported a sensation on the hand; PA_max_ is the value of PA below the motor threshold at which the subject reported a well-defined and conformable sensation. The stimulation duration was settled to 0.5 s.

In the median nerve stimulation phase, during the charge modulation, the PF was fixed at 150 Hz, and the PW was varied in the range of 100–500 μs with a step of 40 μs. At the end of the charge modulation, PW_m_ and PW_m0_ were selected. PW_m_ is a value of PW at which the reported sensation intensity was at least 3, and PW_m0_ is the last value of PW at which the reported sensation intensity was 0.

During the frequency modulation, the PW was settled to PW_m_, and the PF of the stimulus varied in the range of 50–500 Hz with a step of 50 Hz from 50 to 200 Hz and a step of 100 Hz from 200 to 500 Hz. At the end of the frequency modulation, PF_m_ and PF_m0_ were selected. PF_m_ is a value of PF at which the reported sensation intensity is at least 3, and PF_m0_ is the last value of PF at which the reported sensation intensity is 0.

In the ulnar nerve stimulation phase, during the charge modulation, the PF was fixed at 150 Hz, and the PW was varied in the range of 300–600 μs with a step of 40 μs. PW_u_ and PW_u0_ were selected in the same way as described for the charge modulation of median stimulation phase. During the frequency modulation, the PW was PW_u_ and the PF of the stimulus varied from 50 to 600 Hz with analogous median nerve stimulation steps. At the end of this section, PF_u_ and PF_u0_ were selected in the same way as described for the frequency modulation of median nerve stimulation phase.

For the last phase of the mapping protocol, the stimuli parameters should have to be settled for applying the concurrent stimulation. In the charge modulation, the PF was fixed at 150 Hz and the PW varied from PW_m0_ to 500 μs for the median nerve, whereas PW varied from PW_u0_ to 600 μs for the ulnar nerve. At the end of the charge modulation, PW_mc_ and PW_uc_ were selected as the two values of PW for, respectively, the median and ulnar nerve at which the reported sensation intensities are at least 3. In the frequency modulation, the PW were fixed at PW_mc_ and PW_uc_, respectively, for the median and ulnar stimuli, and PF varied in the frequency ranges of median and ulnar nerves.

For all the three stimulation phases, the maximum pulse amplitude and the stimulus duration (0.5 s) was kept constant during both modulation phases.

A correlation analysis was conducted for the results obtained during the charge and frequency modulation of the mapping protocol. For the charge modulation, the correlation and the linear regression between data of the injected charge to the subjects and the referred intensities reported by the subjects were studied. For the frequency modulation, the correlation and the linear regression between the PF of the stimulus and the referred intensities reported by the subjects were studied.

### AMS Strategy

The AMS strategy recreates an apparent movement sensation in the hand of the subject that moves from the fingers innervated by the median nerve to the ones innervated by the ulnar nerve and reverse. AMS is based on the psychological phenomenon called *tactile phi phenomenon*; thus, properly modulating the two stimuli intensities could recreate a slippage sensation. The slippage sensation was delivered by an AMS that flows along the fingers. AMS can be generated by means of three different methods: PAV (Izumi et al., 1988; Rahal et al., 2009), PWV (Pfeifer et al., 2010; Arieta et al., 2011; Seps et al., 2011), and interstimulus delay modulation (ISDM) methods, applied to median, ulnar, and concurrent stimulation phases (Figure 2B).

In the median and ulnar stimulation, the type of sensation elicited on the hand of the subjects was investigated for two different time durations (0.5 and 1 s). In the concurrent stimulation phase, a comparison between the PAV and PWV methods in median–ulnar (MU) and ulnar–median (UM) directions in the two different time durations (0.5 and 1 s) was carried out. Moreover, the three methods were further investigated to recreate the AMS on the whole hand, and their effects were compared.

In the PAV method, the pulse amplitude of each subject was modulated in five steps, from PA_max_ to PA_min_, identified by the subject for each nerve in the mapping protocol. The PW and the PF were kept constant at PW_m_/ PW_u_ for the median/ulnar nerve and at PF_m_/PF_u_ for the median/ulnar nerve.

The PWV method consisted in modulating the pulse width in five steps from PW_m_/PW_u_ to PW_min_ of the median and ulnar nerve. This last value was identified stimulating the subject with PA_max_, PF_m_/PF_u_ and decreasing PW from PW_m_/PW_u_ with a step of 20 μs.

The ISDM concerned the modulation of the delay between the two signals sent to the two nerves, keeping constant the PA_max_, the PW_m_/PW_u_, and the PF_m_/PF_u_. The delay was varied in the range of 0–0.5 s with a step of 0.1 s.

During the concurrent stimulation, in order to generate the AMS from the median region of the hand to the ulnar one with the PAV and PWV methods, a signal with decreasing PA or PW was sent to the median nerve, and one with increasing PA or PW was sent to the ulnar nerve. The signals sent to the nerves were inverted to recreate the AMS in the opposite direction. Applying the ISDM method, the movement of the sensation in the median–ulnar direction was recreated by delaying the ulnar signal; for the opposite direction, the median signal was delayed.

For the single nerve stimulation phases, the subject reported the elicited sensation and indicated the preference between the PAV and PWV methods for each direction.

In the concurrent stimulation, the subject was asked to describe the perceived sensation and indicate the perceived movement direction. A success rate (SR) was introduced in order to evaluate if the subject was able to correctly discriminate the movement direction on the hand. The SR was defined as the number of times the subjects discriminate the movement direction out of the all trials for each movement direction. Moreover, for each trial, the subject was asked to classify the three methods resembling the ranking of preference among them.

Two different statistical significance analyses were conducted: one on the success rate and the other one on the ranking preference of the three methods through the application of the Wilcoxon–Mann–Whitney test with Bonferroni correction (level of significance of *P* < 0.016).

## Results

### Mapping Protocol

The maximum current amplitude delivered to the participants was specific for each subject; the mean value ± SD among the subjects is 2.9 ± 0.7 mA for the median nerve and 2.6 ± 1.0 mA for the ulnar nerve.

**FIGURE 3:**
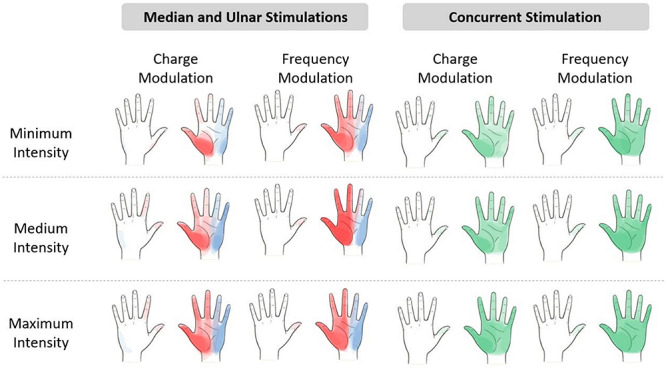
Regions reported by the subjects after the median (red), ulnar (blue), and concurrent (green) stimulations. The regions indicated by the nine subjects have been overlapped in a hand map, which is divided into minimum, medium, and maximum intensity of the electrical stimuli. The areas depicted with a more vivid color indicate the regions with a higher number of occurrences than the others more reported by the subjects. The levels of intensity are the range of values of pulse width (PW) and pulse frequency (PF) provided during the stimuli. Minimum intensity for the median nerve is PW = [220;300] μs and PF = [50;100] Hz and for the ulnar nerve is PW = [380;420] μs and PF = [50;100] Hz. Medium intensity for the median nerve is PW = [340;420] μs and PF = [150;300] Hz and for the ulnar nerve is PW = [460;500] μs and PF = [150;300] Hz. Maximum intensity for the median nerve is PW = [460;500] μs and PF = [400;500] Hz and for the ulnar nerve is PW = [540;580] μs and PF = [400;600] Hz.

The referred sensations in the hand were indicated by the subjects on a map representing the dorsal and palmar side of the hand on the graphic user interface. The regions indicated by the nine subjects were overlapped in order to obtain a single picture indicating the mean region reported by the subjects for three different level of the stimulus intensity. The elicited regions experienced during the charge and frequency modulation of the median, ulnar, and concurrent stimulations are represented in Figure 3. It is worth noticing that, as expected from the literature (D’Anna et al., 2017, 2019; Vargas et al., 2019), the median and the ulnar stimulation elicited, respectively, the regions of the hand innerved by the median and the ulnar nerve, and the concurrent stimulation was able to elicit sensations almost on the whole hand. The areas depicted with a more vivid color indicate the regions reported with a higher number of occurrences than the others.

The extension of the elicited region proportionally increased with the stimulus intensity due to the increase in PW and PF in the charge and frequency modulation. Moreover, the regions reported during the median and ulnar stimulation almost summed up during the concurrent stimulation; these results confirmed the ones obtained in the literature (D’Anna et al., 2017; Vargas et al., 2019).

Tables 1–3 show the naturalness, the depth, the pain, and the quality of the sensation of each trial of the mapping protocol for the median, ulnar, and concurrent stimulation for both charge and frequency modulation.

**TABLE 1:** Characteristics of the elicited sensations for the charge modulation and frequency modulation of the median nerve stimulation.

Charge modulation							
Naturalness (57)		Depth (57)		Pain (57)		Quality (72)	
Natural	21	Superficial	42	0 (No pain)	57	Nothing	15
Almost natural	16	Deep	2	1,2,3	0	Tingling	43
Possibly natural	1	Both	13	4,5,6	0	Vibration	7
Almost unnatural	5			7,8,9	0	Tingling and vibration	6
Unnatural	14			10 (Most pain)	0	Others	1

**Frequency modulation**							
**Naturalness (61)**		**Depth (61)**		**Pain (61)**		**Quality (63)**	

Natural	12	Superficial	40	0 (No pain)	56	Nothing	2
Almost natural	20	Deep	10	1,2,3	5	Tingling	10
Possibly natural	17	Both	11	4,5,6	0	Vibration	19
Almost unnatural	8			7,8,9	0	Tingling and vibration	29
Unnatural	4			10 (Most pain)	0	Others	3

*The total number of trials for the charge modulation (72) and the frequency modulation (63) in naturalness, depth, and pain differ from that of quality because the participant did not always feel a sensation in response to stimulus.*

**TABLE 2:** Characteristics of the elicited sensations for the charge modulation and frequency modulation of the ulnar nerve stimulation.

Charge modulation							
Naturalness (44)		Depth (44)		Pain (44)		Quality (54)	
Natural	12	Superficial	32	0 (No pain)	38	Nothing	10
Almost natural	8	Deep	11	1,2,3	6	Tingling	27
Possibly natural	8	Both	1	4,5,6	0	Vibration	3
Almost unnatural	4			7,8,9	0	Tingling and vibration	13
Unnatural	12			10 (Most pain)	0	Others	1

**Frequency modulation**							
**Naturalness (69)**		**Depth (69)**		**Pain (69)**		**Quality (72)**	

Natural	6	Superficial	48	0 (No pain)	59	Nothing	3
Almost natural	27	Deep	11	1,2,3	10	Tingling	8
Possibly natural	15	Both	10	4,5,6	0	Vibration	23
Almost unnatural	7			7,8,9	0	Tingling and vibration	37
Unnatural	14			10 (Most pain)	0	Others	1

*The total numbers of trials for the charge modulation (54) and the frequency modulation (72) in naturalness, depth, and pain differ from that of quality because the participant did not always provide a sensation in response to stimulus.*

**TABLE 3:** Characteristics of the elicited sensations for the charge modulation and frequency modulation of the concurrent stimulation.

Charge modulation							
Naturalness (50)		Depth (50)		Pain (50)		Quality (59)	
Natural	11	Superficial	43	0 (No pain)	45	Nothing	9
Almost natural	19	Deep	2	1,2,3	5	Tingling	23
Possibly natural	5	Both	5	4,5,6	0	Vibration	1
Almost unnatural	7			7,8,9	0	Tingling and vibration	25
Unnatural	8			10 (Most pain)	0	Others	1

**Frequency modulation**							
**Naturalness (64)**		**Depth (64)**		**Pain (64)**		**Quality (65)**	

Natural	15	Superficial	48	0 (No pain)	56	Nothing	1
Almost natural	9	Deep	8	1,2,3	8	Tingling	3
Possibly natural	15	Both	8	4,5,6	0	Vibration	12
Almost unnatural	13			7,8,9	0	Tingling and vibration	44
Unnatural	12			10 (Most pain)	0	Others	5

*The total number of trials for the charge modulation (59) and the frequency modulation (65) in naturalness, depth, and pain differ from that of quality because the participant did not always provide a sensation in response to stimulus.*

The naturalness of the sensations during the charge modulation of the three stimulation phases was generally perceived natural or almost natural (49% of the trials where the subjects reported a sensation), possibly natural (23%), and almost unnatural and unnatural (28%). During the frequency modulation, the sensations were perceived natural or almost natural for the 46%, possibly natural for the 24%, and almost unnatural and unnatural for the 30%.

Single and concurrent nerve stimulation, during charge and frequency modulation, evoked mostly superficial and painless sensations. However, some subjects reported to feel pain, assigning a value of 1, 2, or 3, especially for the frequency modulation of the ulnar and concurrent stimulations. Nevertheless, only 9% of the total stimulation trials delivered to the subjects produced a pain sensation. In general, the pain was described by the subjects as annoying sensations on the hand or on the forearm.

When the first three stimuli of PW (100, 140, and 180 μs) were applied during the charge modulation of the median nerve, all the subjects reported not to feel any sensations on the hand; the same happened for the first two value of PW (300 and 340 μs) for the ulnar nerve. In Tables 1, 2 and for the other results of the paper, these stimuli were not reported from the total amount of charge modulation trials.

During the charge modulation, 21% of the total number of trials of the median stimulation (72) did not elicit any sensation on the subjects, 19% of the total number of trials of the ulnar stimulation (54), and 15% of the total number of trials of the concurrent stimulation (59). In the remaining trials, the subjects reported a sensation of tingling, vibration, and a combination of them (tingling and vibration). These three sensations were prevalent with respect to the others: they were reported with a percentage of 78, 80, and 83%, respectively, for the median, ulnar, and concurrent stimulations.

Moreover, during frequency modulation, 3% of the total number of trials of the median stimulation (63) did not elicit any sensation on the subjects, 4% of the total number of trials of the ulnar stimulation (72), and 2% of the total number of trials of the concurrent stimulation (65). As it can be seen, the number of times when the subjects did not report any sensation during the frequency modulation is less than the charge one. In the remaining trials, the subjects reported a sensation of tingling, vibration, and a combination of them (tingling and vibration). These resulted in the main qualities reported by the subjects with a percentage of 92, 94, and 91%, respectively, for the median, ulnar, and concurrent stimulations.

**FIGURE 4:**
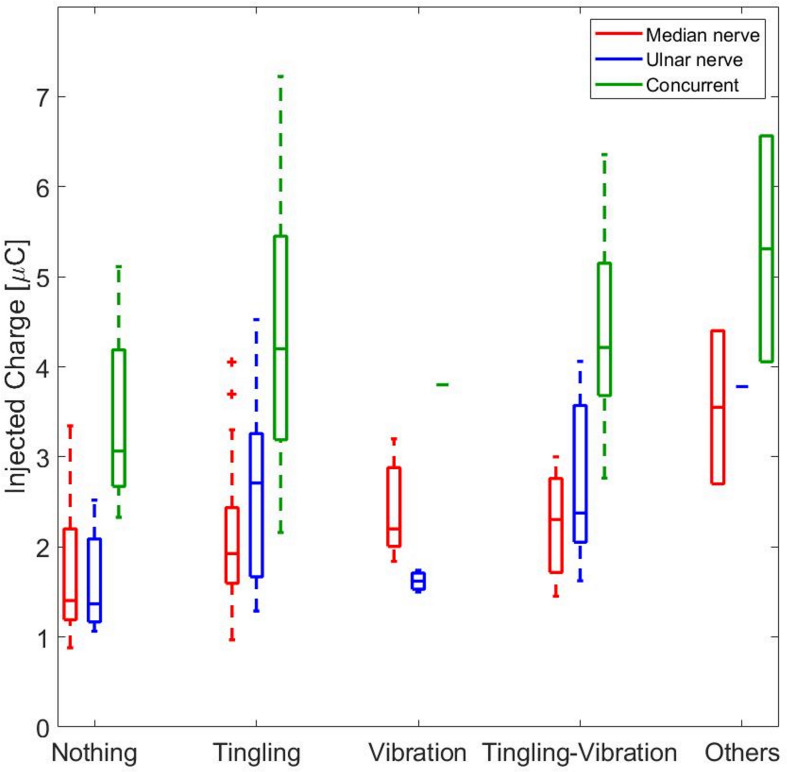
The red boxes represent the median values of the injected charge necessary to induce a specific type of quality during the median stimulation. The blue and the green boxes represent the same results obtained, respectively, during the ulnar and concurrent stimulations. The + signs represents the outliers of the median for each box.

The relation between the quality of the referred sensation and the injected charge was analyzed. Figure 4 shows this relation for the median, ulnar, and concurrent stimulations. The thresholds of the qualities of the elicited sensations are shown when the injected charge was modulated. It seems to have a slight increase in the strength of the sensation when there is a higher quantity of charge. The first type of quality perceived by the subjects was the tingling, and it occurred when 2, 2.7, and 4 μC was applied on the skin for the median, ulnar, and concurrent stimulations, respectively.

**FIGURE 5:**
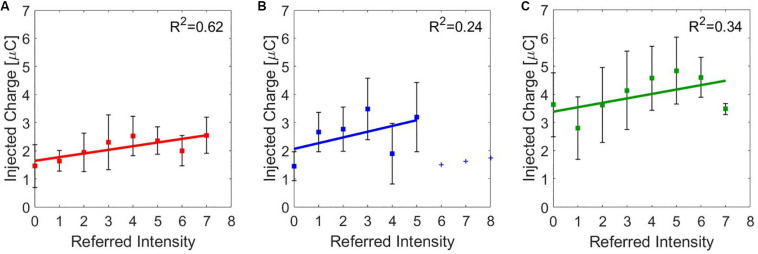
Linear regressions of the median value (±SD, standard deviation) of the injected charge for the referred intensities reported by the subjects during charge modulation of the **(A)** median, **(B)** ulnar, and **(C)** concurrent stimulations. *R*^2^ is the coefficient of determination, which describes the goodness of the linear regression.

The correlation between the median value of the injected charge in the subjects was analyzed during the three stimulation and the correspondence referred intensities. They have a moderate correlation for the median nerve (*ρ* = 0.5798, Pearson coefficient) and a weak one for the ulnar nerve (*ρ* = 0.3205, Pearson coefficient) and for the concurrent stimulation (*ρ* = 0.3813, Pearson coefficient). Then, a linear regression was conducted on the three data set in order to determine the coefficients of determination (*R*^2^). In Figure 5, the linear regressions are represented: for the median nerve, *R*^2^ = 0.62; for the ulnar nerve and for the concurrent stimulation, *R*^2^ = 0.24 and *R*^2^ = 0.34, respectively.

Moreover, it is relevant that the charge needed to stimulate the ulnar nerve and both nerves simultaneously, with equal reported sensation intensity, was higher with respect to the median nerve (Figure 5). The ulnar nerve could be anatomically located more in depth in the segment of the forearm where the superficial electrodes were placed; thus, more charge was needed to obtain the same type and intensity of sensation elicited on the areas innervated by the median nerve. During concurrent stimulation, highest values of charge were injected because the sum of the injected charge of the two single nerves was delivered.

**FIGURE 6:**
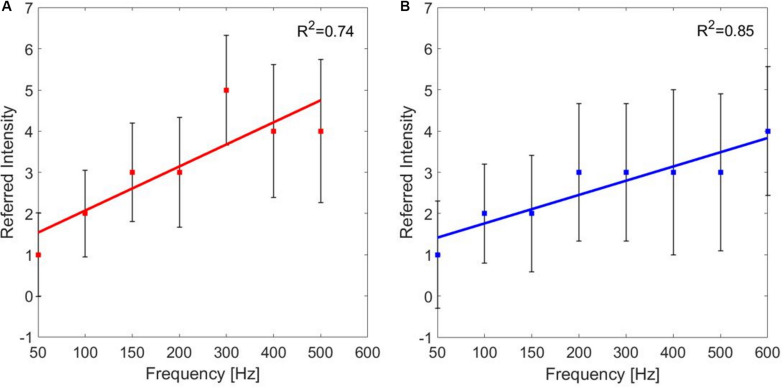
Linear regressions of the median value (±SD, standard deviation) of the referred intensities reported by the subjects for each pulse frequency (PF) of frequency modulation of the **(A)** median and **(B)** ulnar stimulations. *R*^2^ is the coefficient of determination, which describes the goodness of the linear regression.

For each PF, the median values of the referred intensities reported by the subjects during the frequency modulation were calculated. The correlation between the PF of the stimulus and the referred intensities for the median and the ulnar stimulations was studied. The correlation between PF and referred intensities is moderate for both the median and the ulnar nerve (*ρ* = 0.6771 and *ρ* = 0.6015, Pearson coefficient). Then, the linear regressions of the two data set were studied: for both stimulation phases are *R*^2^ = 0.74 and *R*^2^ = 0.85, respectively, for the median and ulnar nerve. The median of the referred intensities reported from the nine subjects increases with frequency, as it is shown in Figure 6.

### AMS Strategy

The aim of the AMS protocol was recreating a sensation of movement in the hand of the subjects. Primarily, the PAV and the PWV were compared during single nerve stimulations. Then, the three methods (i.e., PAV, PWV, and ISDM) were compared during concurrent stimulation.

The single nerve stimulations during the AMS protocol did not revealed any substantial results. The subject perceived a rapid moving sensation starting from the forearm, in correspondence with the electrodes, and reaching the hand and vice versa.

During the concurrent stimulation, the subject had to indicate the perceived direction of the movement elicited during the AMS protocol. Table 4 reports the SRs obtained during concurrent stimulation of the AMS protocol. The results show that the SR of AMS for the median–ulnar direction is 0.83 for PAV method, 0.89 for PWV method, and 0.98 for the ISDM method, whereas, for the ulnar–median direction, the SR is 0.78 for PAV method, 0.89 for PWV method, and 0.98 for the ISDM method (see Table 4). This means that, for each movement direction, the SR of ISDM method is higher than that of PAV and PWV for recreating an AMS in the hand that easily allows distinguishing the movement direction. It is a reliable technique also because all the subjects were able to understand the AMS with this method. Subject 9 in fact did not understood the moving sensation with the first two methods.

**TABLE 4:** Comparison between success rate (SR) obtained, applying the three methods for each movement directions (MU, median–ulnar; UM, ulnar–median).

	PAV	PWV	ISDM
MU	0.83	0.89	0.98
UM	0.78	0.89	0.98

Table 5 shows the percentage of preferences expressed by the subjects for the two stimulus durations for PAV and PWV and for the delays of the ISDM method.

**TABLE 5:** Percentage of preferences expressed by the subjects of the two different time duration of the stimulus (0.5 or 1 s) for each movement directions (MU, median–ulnar; UM, ulnar–median) for the pulse amplitude variation (PAV) and pulse width variation (PWV).

	PAV		PWV		ISDM					
Stimulus Duration (s)	0.5	1	0.5	1	Delays (s)	0.1	0.2	0.3	0.4	0.5
MU	67%	33%	44%	56%	MU	11%	33%	33%	11%	11%
UM	33%	67%	22%	78%	UM	11%	22%	22%	33%	11%

*For the ISDM, the percentages of preferences expressed by the subjects of delay between the two signals sent to the two nerves (0.1–0.5 s with a step of 0.1 s) are reported for each movement directions.*

There was not a clear preference between the two different time durations of the stimuli for the PAV and the PWV, as it is reported in Table 5. Among the delays for the median–ulnar direction, the highest percentage of preference was equal for 0.2 and 0.3 s (both 33% of preference); for the ulnar–median direction, the 33% preference was for the 0.4-s delay.

**FIGURE 7:**
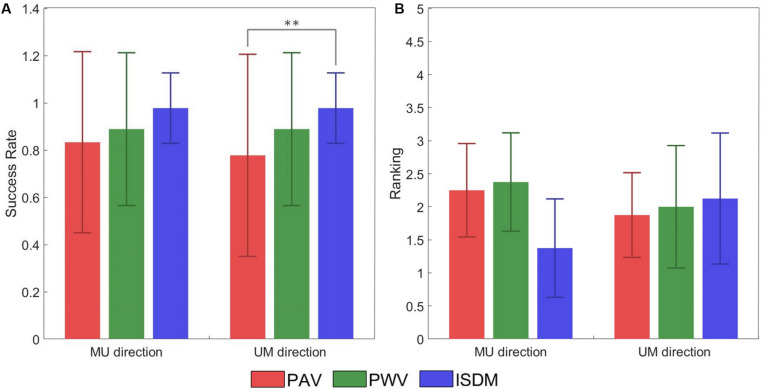
**(A)** Success rate (±SD, standard deviation) of the discrimination of the movement direction of each subject for each method for both median–ulnar (MU) and ulnar–median (UM) directions. The comparative analysis (Wilcoxon–Mann–Whitney test with Bonferroni correction) reports a statistically significant difference in terms of success rate (SR) between pulse amplitude variation (PAV) and ISDM for the UM direction (*P*_PAV–ISDM_ = 0.0089). No statistically significant differences were reported for the other comparisons: for the MU direction *P*_PAV–ISDM_ = 0.0367, *P*_PAV–PWV_ = 0.6536, and *P*_PWV–ISDM_ = 0.1432; for the UM direction, *P*_PAV–PWV_ = 0.3912 and *P*_PWV–ISDM_ = 0.1432. **(B)** Mean of the ranking position (± SD, standard deviation) for each method for both MU and UM direction. Low mean values indicate that the subjects ranked the method in high positions like 1 or 2. The comparative analysis (Wilcoxon–Mann–Whitney test with Bonferroni correction) reports no statistically significant differences in terms of ranking preference among PAV, PWV, and ISDM: for the MU direction, *P*_PAV–ISDM_ = 0.0340, *P*_PAV–PWV_ = 0.8858, and *P*_PWV–ISDM_ = 0.0297; for the UM direction, *P*_PAV–ISDM_ = 0.7630, *P*_PAV–PWV_ = 0.9992, and *P*_PWV–ISDM_ = 0.9184. Statistically significant differences (*P* < 0.0016) are depicted by asterisks.

Figure 7A represented the SR of the discrimination of the movement direction of each subject for each method for both MU and UM directions. The statistical analysis (Wilcoxon–Mann–Whitney test with Bonferroni correction) pointed out a significant difference between PAV and ISDM for the UM direction. The *P*-value *P*_M1–M2_ means the *P*-value of the comparison between two methods, M1 and M2. For the MU direction: *P*_PAV–ISDM_ = 0.0367, *P*_PAV–PWV_ = 0.6536, and *P*_PWV–ISDM_ = 0.1432. For the UM direction: *P*_PAV–ISDM_ = 0.0089, *P*_PAV–PWV_ = 0.3912, and *P*_PWV–ISDM_ = 0.1432.

At the end of the AMS protocol, the three methods were compared, and each subject expressed a preference among them. Figure 7B reported the ranking position for each method for both MU and UM direction. Low mean values indicate that the subjects ranked the method in high positions like 1 or 2. For the MU direction of movement, seven subjects out of nine reported ISDM as the best method. These results show that ISDM method is indicated by the subjects as the favorite method for recreating a well-defined and conformable AMS for the MU direction. For the UM direction, there was not a clear preference for one of the three methods. The comparative analysis (Wilcoxon–Mann–Whitney test with Bonferroni correction) reported no statistically significant differences in terms of ranking position among PAV, PWV, and ISDM; therefore, there is no significant preference of the three methods. For the MU direction: *P*_PAV–ISDM_ = 0.0340, *P*_PAV–PWV_ = 0.8858, and *P*_PWV–ISDM_ = 0.0297. For the UM direction: *P*_PAV–ISDM_ = 0.7630, *P*_PAV–PWV_ = 0.9992, and *P*_PWV–ISDM_ = 0.9184.

## Discussion

This study wanted to investigate the feasibility of using a non-invasive interface based on TENS in a closed-loop device for restoring tactile feedback in terms of forces and slippage. Static tactile sensations and an AMS were recreated in the hand of nine healthy subjects to reproduce sensations occurring during object grasping and manipulation (where the contact between hand and object can be dynamic). An experimental protocol composed of a mapping protocol and AMS protocol was developed.

The mapping protocol allowed characterizing the type of referred sensation in term of naturalness, depth, pain, intensity, and quality. At the end of each trial, for each subject, a hand map was reconstructed where the elicited regions were pointed out. During the charge modulation, the delivered sensations were mostly described by the subjects as an almost natural and superficial tingling. While in the frequency modulation, the sensation was mainly perceived as tingling/vibration. Moreover, the increase in the injected charge intensified the sensation, through the variation of the quality, the referred intensity, and the elicited regions. The correlation analysis for the charge modulation showed that the correlation between the referred intensity and the injected charge is moderate for the median stimulation and weak for the ulnar and concurrent stimulations. For the frequency modulation, the correlation between the PF of the stimulus and the referred intensity is moderate for both nerves.

The obtained results from the mapping protocol matched with the literature background (Chai et al., 2015; Osborn et al., 2017; Shin et al., 2018; Vargas et al., 2019). They strengthened the achievements of TENS studies carried out until now. The data collected from the mapping protocol represent fundamental information for further investigations. In this case, they were used to extend the experimental protocol in order to recreate more complex sensations on the hand, enlarging the literature. In particular, they suggest that there is a common and shared way to characterize sensations, for extrapolating subject-dependent information for specific applications. Further analysis could be the comparison within the subjects among sensation characteristics, such as naturalness or accuracy, or between stimulation methods (charge or frequency modulation) in order to evaluate the ability to perceive different levels of referred intensity and assess the subject acceptance of TENS technique. Moreover, the shifting of the user’s tissues in the stump–socket interface from normal movements leads to a variation of the level of impedance. Consequently, this could produce a change in the level of intensity of the referred sensation and/or in the sensation itself. Nevertheless, this would not affect the goodness of TENS since this problem could be overcome by a remodulation of stimulation parameters. In future studies, it will be useful to verify this condition by changing the stimulation parameters in order to compensate the referred sensation perception.

On the other hand, the AMS experimental protocol allowed eliciting an AMS on eight subjects out of nine through three different methods. Only one of the subjects was not able to feel the AMS with the PAV and PWV methods.

In general, the three strategies were able to reach the intended target of discriminating the movement direction: the SR of AMS for the median–ulnar direction is 0.83 for PAV method, 0.89 for PWV method, and 0.98 for the ISDM method, whereas for the ulnar–median direction, the SR is 0.78 for PAV method, 0.89 for PWV method, and 0.98 for the ISDM method (Table 4). The comparative analysis (Wilcoxon–Mann–Whitney test with Bonferroni correction) reported a statistically significant difference in terms of SR of movement direction discrimination between PAV and ISDM methods for the UM direction (*P* < 0.016, see Figure 7A). The other comparisons did not show significantly differences.

The mean of the ranking position of the three methods for the MU direction is low for the ISDM method, so more subjects indicated it as the favorite method for eliciting a well-defined moving sensation in the hand. The Wilcoxon–Mann–Whitney test with Bonferroni correction did not highlight a statistically significant difference in terms of ranking positions among the three methods (Figure 7B).

In this study, AMS protocol permitted to create a moving sensation on the hand of nine healthy subjects in a non-invasive way with the use of TENS. Moreover, TENS guaranteed a somatotopical approach, which recreate a sensation to the corresponding location of missing limb in a physiologically natural way. TENS studies focused on functional tasks in which the objects in contact with the hand is stable. For the first time, it was possible to induce not a static sensation on the hand but a moving one for replicating events that could occur unexpectedly, like slippage. AMS candidates itself as novel tool for feedback restoration during more complex tasks, in which the object is not fixed. Moreover, by this study, high SRs were obtained with healthy subjects, suggesting that it could reasonable to validate this strategy on amputees.

## Conclusion

The experiments confirmed the good potential of recreating slippage sensations by means of an AMS protocol in the hand via TENS. The AMS protocol is a reliable technique that can elicit a moving sensation that is easy to distinguish, especially through the application of the ISDM method. It is a promising technique because it could recreate an AMS on the subject fingers that is assimilable to the variation of the contact point of the hand during object manipulation tasks.

In the future, AMS needs to be investigated more in depth in order to elicit a movement sensation passing through the fingers of the hand. In fact, not all the subjects were able to discriminate this type of transition during the AMS; some of them only perceived the sensation moving between the two regions innervated by the median and the ulnar nerves.

After the feasibility study conducted in this paper for recreating AMS by the means of TENS in nine healthy subjects, future improvements will be testing the proposed approach with amputees, whose nerves could have undergone a reorganization into the tissues.

Up to the present, this technique was never studied to recreate a moving sensation in the hand, but it was only examined on lower limb during gait analysis and posture control (Rahal et al., 2009; Pfeifer et al., 2010; Seps et al., 2011; Pagel et al., 2016). Thanks to the promising results, this technique could be integrated in the closed loop of a prosthetic system in order to elicit the moving sensation of an object among the prosthetic fingers, as in the slippage events, and provide the user with information about manipulation forces and slippage event during grasp control.

## Data Availability Statement

The datasets generated for this study are available on request to the corresponding author.

## Ethics Statement

The experimental protocol was approved by the local Ethical Committee (Comitato Etico Università Campus Bio-Medico di Roma) and complied with the Declaration of Helsinki. All subjects gave written informed consent in accordance with the Declaration of Helsinki.

## Author Contributions

AS analyzed the literature, designed the proposed approach, acquired and analyzed the experimental data, and wrote the manuscript. AD analyze the literature, designed the proposed approach, analyze the experimental data, and contributed to wrote the manuscript. FT contributed to analysis of the literature, to design of the proposed approach, and to acquisition of the experimental data. AC contributed to the design of the proposed approach and of the experimental setup, wrote the manuscript, and supervised the study. LZ designed the manuscript and supervised the study. All the authors read and approved the manuscript.

## Conflict of Interest

The authors declare that the research was conducted in the absence of any commercial or financial relationships that could be construed as a potential conflict of interest.
